# The effect of perioperative tranexamic acid (TXA) in patients with calcaneal fractures: a meta-analysis and systematic review of randomized controlled trials

**DOI:** 10.1186/s13018-023-03924-0

**Published:** 2023-07-12

**Authors:** Xiumei Tang, Kai Li, Fuyuan Zheng, Yue He, Yang Yang, Duan Wang

**Affiliations:** 1 Department of Respiratory and Critical Care Medicine, Med-X Center for Manufacturing, Frontiers Science Center for Disease-Related Molecular Network, West China Hospital, West China School of Medicine, Sichuan University, Chengdu, 610041 Sichuan People’s Republic of China; 2 Health Management Center, General Practice Medical Center, West China Hospital, Sichuan University/Institute of Hospital Management, West China Hospital, Sichuan University, Chengdu, 610041 People’s Republic of China; 3 Department of Pulmonary and Critical Care Medicine, Chengdu Third People’s Hospital, Southwest Jiaotong University, Chengdu, 610031 Sichuan People’s Republic of China; 4 Department of Respiratory Medicine, The People’s Hospital of Pujiang County, Chengdu, 611630 Sichuan People’s Republic of China; 5 Department of Undergraduate Students, West China School of Medicine, Sichuan University, Chengdu, 610041 Sichuan People’s Republic of China; 6 Department of Orthopaedics, West China Hospital, Sichuan University/West China School of Nursing, Sichuan University, Chengdu, 610041 People’s Republic of China; 7 State Key Laboratory of Oral Diseases, National Clinical Research Center for Oral Diseases, West China Hospital of Stomatology, Sichuan University, Chengdu, People’s Republic of China; 8 Department of Orthopedics, West China Hospital/West China School of Medicine, Sichuan University, Chengdu, 610041 People’s Republic of China

**Keywords:** Tranexamic acid, Calcaneal fractures, Open reduction and internal fixation, Meta-analysis and systematic review, Randomized controlled trials

## Abstract

**Background:**

Calcaneal fractures are a common orthopedic disease, account for approximately 2% of all bone fractures, and represent 60% of fractures of tarsal bones. Tranexamic acid (TXA) is a synthetic antifibrinolytic drug that competitively blocks the lysine-binding sites of plasminogen, plasmin, and tissue plasminogen activator, delaying fibrinolysis and blood clot degradation. However, the effect of TXA on patients with calcaneal surgery remains controversial. Our objective was to evaluate the effectiveness of TXA in calcaneal fractures surgeries.

**Methods:**

The electronic literature databases of Pubmed, Embase, and Cochrane library were searched in December 2022. The data on blood loss, the stay in the hospital, the duration of surgery, hemoglobin, hematocrit, platelet count, prothrombin time, activated partial thromboplastin time, and wound complication were extracted. The Stata 22.0 software was used for the meta-analysis.

**Results:**

Four randomized controlled studies met our inclusion criteria. This meta-analysis showed that TXA significantly reduced postoperative blood loss during the first 24 h (*p* < 0.001), improved the level of hemoglobin (*p* < 0.001) and hematocrit (*p* = 0.03), and reduced the risk of wound complications (*p* = 0.04). There was no significant difference between the two groups regarding total and intraoperative blood loss, hospital stay, duration of surgery, platelet count, activated partial thromboplastin time, and prothrombin time.

**Conclusion:**

TXA significantly reduced blood loss during the first 24 h postoperatively, improved the level of hemoglobin and hematocrit, and reduced the risk of wound complications. Given the evidence, TXA can be used in patients with calcaneal fractures and had the potential benefit of blood reduction.

**Protocol registration:**

The protocol was registered in PROSPERO (registration No. CRD42023391211).

## Highlights

- As we know, this is the first meta-analysis to focus on the safety and effectiveness of tranexamic acid (TXA) in the fields of calcaneal fracture surgeries which include percutaneous screw fixation, open reduction, and internal fixation.
- Our study was prospectively registered on the PROSPERO website. The search was conducted without any language restrictions and the results were reported according to the PRISMA checklist. The quality of evidence was assessed using the Grading Recommendations Assessment, Development, and Evaluation (GRADE) system. Thus, we yield a comprehensive group of eligible studies and dependable results for our conclusion.
- In this meta-analysis and systematic review, TXA significantly reduced blood loss during the first 24 h postoperatively, improved the levels of hemoglobin and hematocrit, and reduced the risk of wound complications. In view of the evidence, TXA can be safely used in patients with calcaneal fractures and had the potential benefit of blood reduction.

## Background

The incidence of calcaneal fractures is the highest among all tarsal fractures and accounts for approximately 2% of body fractures [1]. It commonly occurs during a high-energy event, such as a car crash or a fall from a ladder and is painful and disabling. Surgeries (e.g. percutaneous screw fixation, open reduction, and internal fixation) are recommended if the bones are displaced [2]. The calcaneus is a spongy cancellous bone with a rich blood supply, and it easily forms a cavity after fracture surgeries with a rich blood supply of its surrounding soft tissues [3]. The traditional lateral “L” approach is a classic approach for managing calcaneal fractures with the drawbacks of excessive surgical trauma and significant blood loss, which may increase the opportunity of infection and delay wound healing [4]. Thus, blood management after calcaneal fractures are of vital importance.

Tranexamic acid (TXA) is a synthetic antifibrinolytic agent that competitively blocks the lysine-binding sites of plasminogen and tissue plasminogen activators, thereby delaying the degradation of fibrinolytic and blood clots [5]. So far, numerous randomized controlled trials (RCTs) and meta-analyses have discussed TXA in various surgery types and reported significant effects. TXA was demonstrated to be a safe and effective choice in general surgeries, joint surgeries [6], cardiac surgeries [7], spine surgeries [8], neuro surgeries, gynecologic surgeries, and other types of surgeries [9]. Meta-analysis showed that TXA reduced blood loss irrespective of the type of surgery [10]. In calcaneal fracture surgery, however, the effectiveness of TXA is still unknown. Recently, trials concerning the safety and effectiveness of TXA in patients with calcaneal fractures have been published [11]. However, the results were controversial, and synthesized evidence was lacking.

To provide a comprehensive review of existing evidence, we performed a systematic review and meta-analyses to demonstrate the efficacy and safety of TXA in patients with calcaneal fractures surgeries and provide evidence for clinical application. Our primary outcome was perioperative blood loss, and secondary outcomes were perioperative complications.

## Methods

### Search strategy

Our review followed the Preferred Reporting Items for Systematic Reviews and Meta-analyses (PRISMA) guidelines, and the protocol was registered in PROSPERO (registration No *CRD42023240303*). Two independent researchers searched Medline, Embase, Web of Science, and Cochrane databases updated to 28 February 2023 for meta-analyses. Two independent researchers conducted the literature searches using the search strategy of (“calcaneal fracture” [Mesh] or “calcaneus” [Mesh]) and (“Tranexamic acid” [Mesh] OR tranexamic acid OR TXA) AND ((randomized controlled trial OR controlled clinical trial OR randomized OR clinical trials). In addition, the reference lists of the previously published randomized trials, review articles, and meta-analyses were manually searched for additional eligible studies. Related articles and reference lists were searched to avoid the original miss of any relevant articles.

### Inclusion criteria

The inclusion criteria followed the PICOs principle. Patients who were diagnosed with displaced intra-articular calcaneal fractures received operative treatment, including open reduction and internal fixation. Interventions were TXA (either topical or intravenous). Control interventions were saline or placebo. And outcomes were one of the predefined outcomes concerning the safety and effectiveness of TXA. We only included RCT studies. We included studies with comparisons of different administration methods and dosages (high versus low dose and any versus none). Included studies must be in accordance with the Declaration of Helsinki and approved by the respective ethics committees. There was no restriction on language and country. Exclusion criteria were no availability of full-text articles, letters, meeting proceedings, and case reports. Two researchers independently screened the titles and abstracts, and articles satisfying the inclusion criteria were accessed for full-text review. They also independently reviewed full-text articles for eligibility. When data was incomplete, the corresponding author was contacted by email and invited to provide additional information. Reference lists of eligible reviews and meta-analyses were searched for additional citations. Any disagreements were resolved by consensus.

### Data extraction

Two researchers independently extracted data from eligible articles based on titles and abstracts and reviewed relevant articles as full text. Disagreements were resolved by discussion and referral to a third author if necessary. Two authors extracted the study characteristics from each included study, including the year of publications, study population, number of participants, name of comparators, the dose of treatment, indication for treatment, name and types of adverse events, and information assessing the risk of bias in the studies.

### Quality assessment

Two authors independently assessed the risk of systematic errors (bias) of the trials included in the meta-analysis according to the Cochrane Handbook, version 6.1. To evaluate the risk of bias in the individual RCTs, we will use the revised uniform criteria of the Cochrane risk-of-bias tool for randomized trials ver. 2 (RoB 2). Risk of bias was rated according to the following domains (1) bias due to randomization, (2) bias due to deviations from indented interventions, (3) bias due to missing data, (4) bias due to outcome measurement, (5) bias due to selection of reported result. Trials adjudicated as having concerns or having a high risk of bias for one or more domains were classified as having an overall high risk of bias.

### Outcomes

The primary outcome was perioperative blood loss, and the secondary outcome was perioperative complications. Our outcomes included i) clinical outcomes: perioperative blood loss, length of hospital stay, duration of surgery, results of blood test (e.i. hemoglobin, hematocrit, platelet count, prothrombin time, and activated partial thromboplastin time), and related complications (wound complication). Subgroup analyses were performed based on the follow-up duration. For instance, the results of perioperative blood loss were subgrouped according to the 0–24 h and 24–48 h periods.

### Statistical analysis

Standard mean difference (SMD) was used for continuous variable statistics, and relative risks (RR) was used for discontinuous variable statistics. We performed random effects analyses using the DerSimonian and Laird estimator. For each included trial, we calculated the relative risks (RRs) with 95% confidence intervals (95% CI) for all outcome measures. Heterogeneity among the trials was explored by inspection of forest plots and calculation of *I*^2^ statistics. Statistical heterogeneity will be evaluated informally from forest plots of the study estimates and more formally using the chi-squared test (*p *value < 0.1 = significant heterogeneity) and *I*^*2*^ statistic (*I*^2^ > 50% = significant heterogeneity). To investigate publication biases, we created funnel plot in which the log RRs were plotted against their standard errors and tested the symmetry of the funnel plots with Egger’s linear regression test. Statistical analyses were performed using Review Manager Software 5 (Review Manager [RevMan] Version 5.4. Copenhagen: The Nordic Cochrane Centre, The Cochrane Collaboration, 2020) and STATA software, V.15.0 (STATA Corporation, College Station, TX, USA).

## Results

### Literature search

**Fig. 1:**
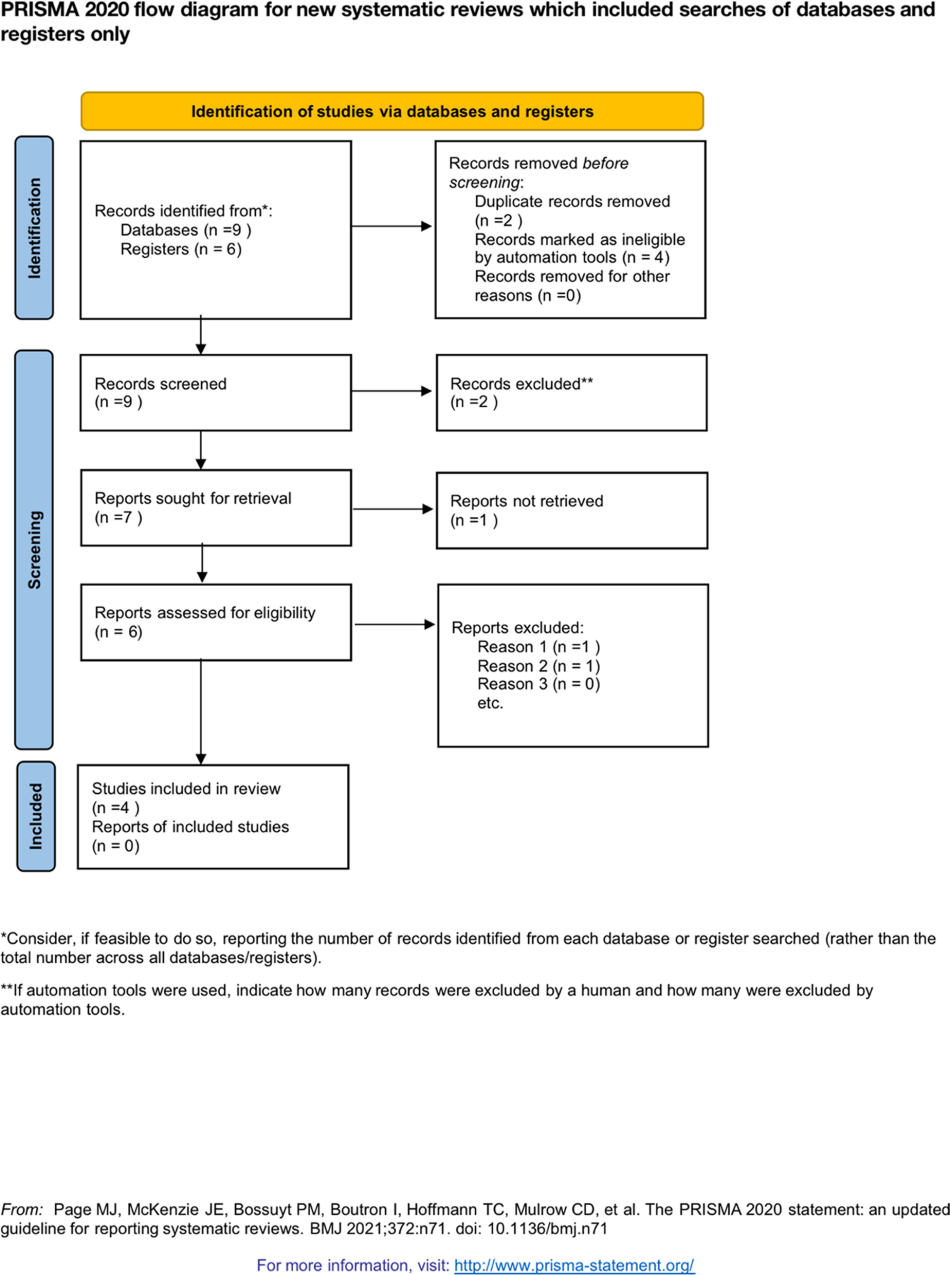
PRISMA flowchart of the study selection

In total, we screened 117 abstracts, of which 43 were eligible for full-text review. 4 trials with 255 participants were eligible for inclusion in the meta-analysis (see Fig. 1).

### Description of included studies

Table 1 summarizes the characteristics of included trials. One trial [4] was published in 2015 and the other 3 trials were published between 2021 and 2022 [12]. The population ranged from 40 to 90. In two trials [13], the administration of TXA was topical, while the other trials [14] were intravenous. The detailed information can be seen in Table 2.

**Table 1:** Baseline characteristic of included studies

Study (year)	Design	Recruit period	No. of patients (n)		Age (years) Mean (SD)		Women, No. (%)		BMI	
			Tranexamic Acid	Control	Tranexamic Acid	Control	Tranexamic Acid	Control	Tranexamic Acid	Control
Huang (2022)	RCT	2017.09–2019.12	20	20	43.9 (11.3)	43.9 (11.3)	1 (5%)	1 (5%)	N/A	N/A
Xie (2015)	RCT	2010.01–2012.12	45	45	43.4 (8.8)	42.6 (9.8)	3 (7.14%)	2 (4.65%)	24.4 (1.7)	24.8 (1.6)
Zhong (2021)	RCT	2014.08–2018.04	34	19	43.1 (8.5)	40.4 (8.9)	7 (25.92%)	4 (26.67%)	23.8 (2.6)	24.0 (2.5)
Wu (2021)	RCT	2020.04–2021.4	36	36	28.44 (1.78)	28.33 (1.79)	16 (44%)	14 (38%)	N/A	N/A

**Table 2:** Confounding information of included studies

Author	Country	Sample (n)	Classification	Women (%)	Administration	Interventions	Controls	Surgeon	Anesthesia	Surgical approach
Huang (2022)	China	40	Sanders: **II–IV**	2 (5)	Topical	(1) Before closure: 80 mL 0.5 g/L TXA; (2) After closing: 20 mL 0.5 g/L TXA;	(1) Before closure: 80 mL 0.9% sodium chloride; (2) After closing: 20 mL 0.9% sodium chloride;	3 well-trained senior surgeons	Intraspinal anesthesia	Lateral extensile incision
Xie (2015)	China	90	Sanders: **II–III**	5 (5.5)	Intravenously	(1) 15 min before surgery: 15 mg/kg mixed in 100 mL of 0.9% sodium chloride solution;	(1) 15 min before surgery: an equal dose of saline only;	4 surgeons	Mixed (conduction anesthesia; epidural anesthesia; general anesthesia)	Standard extended lateral approach to the calcaneum
Zhong (2021)	China	53	Sanders: III–IV	11 (20.75)	Topical	(1) Group A: 20 ml of 10 mg/ml TXA solution; (2) Group B: 20 ml of 20 mg/ml TXA solution;	(1) Group C: 20 ml of saline;	1 surgeon	N/A	open reduction internal fixation (ORIF) via lateral approach with an L-shaped incision
Wu (2021)	China	72	N/A	16 (44)	Intravenous	(1) Before Operation: 1 g .0 g/L TXA;	(1) Before Operation: 1.0 g/L TXA;	N\A	General	"L" shape incision

### Risk of bias in individual trials

Overall, the methodology quality can be seen as moderate. Two trials [15] were deemed to be at low risk of bias and the other two trials were considered moderate [16]. All trials were judged at low risk of bias for the randomization process, and the selection bias in 2 trials [16] was unclear and so as in the performance bias. The detection bias was unclear in all trials (see in Table 3).

**Table 3:** Methodologic quality assessment of included studies (RCT)

Study (year)	Random sequence generation (selection bias)	Allocation concealment (selection bias)	Blinding of participants and personnel (performance bias)	Blinding of outcome assessment (detection bias)	Incomplete outcome data (attrition bias)	Other bias
Huang (2022)	Low risk	Low risk	Low risk	Unclear	Low risk	Low risk
Xie (2015)	Low risk	Low risk	Low risk	Unclear	Low risk	Low risk
Zhong (2021)	Low risk	Unclear	Unclear	Unclear	Low risk	Low risk
Wu (2021)	Low risk	Unclear	Unclear	Unclear	Low risk	Low risk

### Primary outcome

**Fig. 2:**
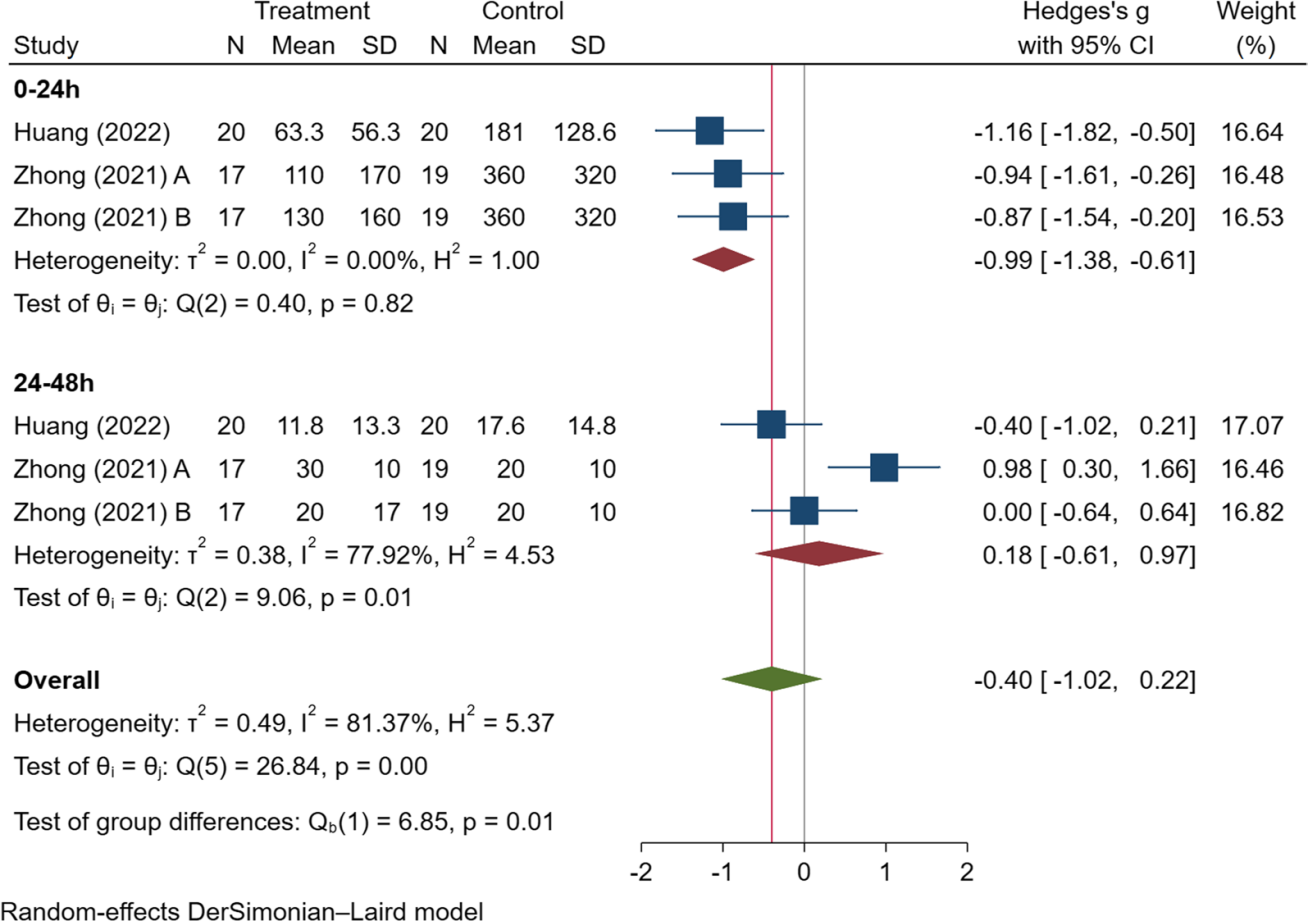
The forest plot on postoperative drainage volume

We found that TXA administration reduced postoperative blood loss within 24 h (SMD = − 0.99 [95% CI − 1.38, − 0.61], *I*^2^ = 0%), moderate certainty of the evidence) (see Fig. 2). However, there was no difference in the intraoperative blood loss (SMD = − 2.78 [95% CI − 7.50, 1.94], *I*^2^ = 98.75%, low certainty of the evidence) (see in Table 4).

**Table 4:** The results of the meta-analysis

Variables	No. of Study	No. of patients intervention	No. of patients control	Pooled data		Heterogeneity	
				SMD/Log RR (95% CI)	*P*	*I*^*2*^ (%)	Ph
*Clinical outcomes*							
Postoperative drainage volume (ml)	3	54	58	− 0.41 (− 1.05, 0.23)	0.21	81.95	< 0.001*
*By subgroup (follow-up time)*							
0–24 h	3	54	58	− 0.99 (− 1.38, − 0.61)	< 0.001*	0	0.82
24–48 h	3	54	58	0.18 (− 0.61, 0.97)	0.65	77.92	0.01*
Intraoperative blood loss (ml)	2	81	81	− 2.78 (− 7.50, 1.94)	0.25	98.75	< 0.001*
Hospital stay (days)	2	65	65	− 1.09 (− 2.44, 0.27)	0.12	91.41	< 0.001*
Duration of surgery (mins)	2	81	81	− 0.38 (− 0.86, 0.10)	0.12	57.79	0.12
*Blood tests*							
Hemoglobin (g/L)	4	99	103	0.77 (0.32, 1.22)	< 0.001*	54.88	0.09
Hematocrit (%)	4	99	103	0.92 (0.12, 1.73)	0.03*	85.32	< 0.001*
Platelet count (10^9/L)	4	99	103	0.04 (− 0.23, 0.32)	0.77	0	0.92
PT (s)	4	99	103	0.17 (− 0.32, 0.65)	0.50	63.42	0.05*
APTT (s)	4	99	103	0.08 (− 0.20, 0.36)	0.57	0	0.64
*Complications*							
Wound complications	2	75	61	− 1.10 (− 2.17, − 0.02)	0.04*	0	0.52

*APTT* activated partial thromboplastin time, *PT* Prothrombin time. * indicates statistically difference

### Secondary outcome

**Fig. 3:**
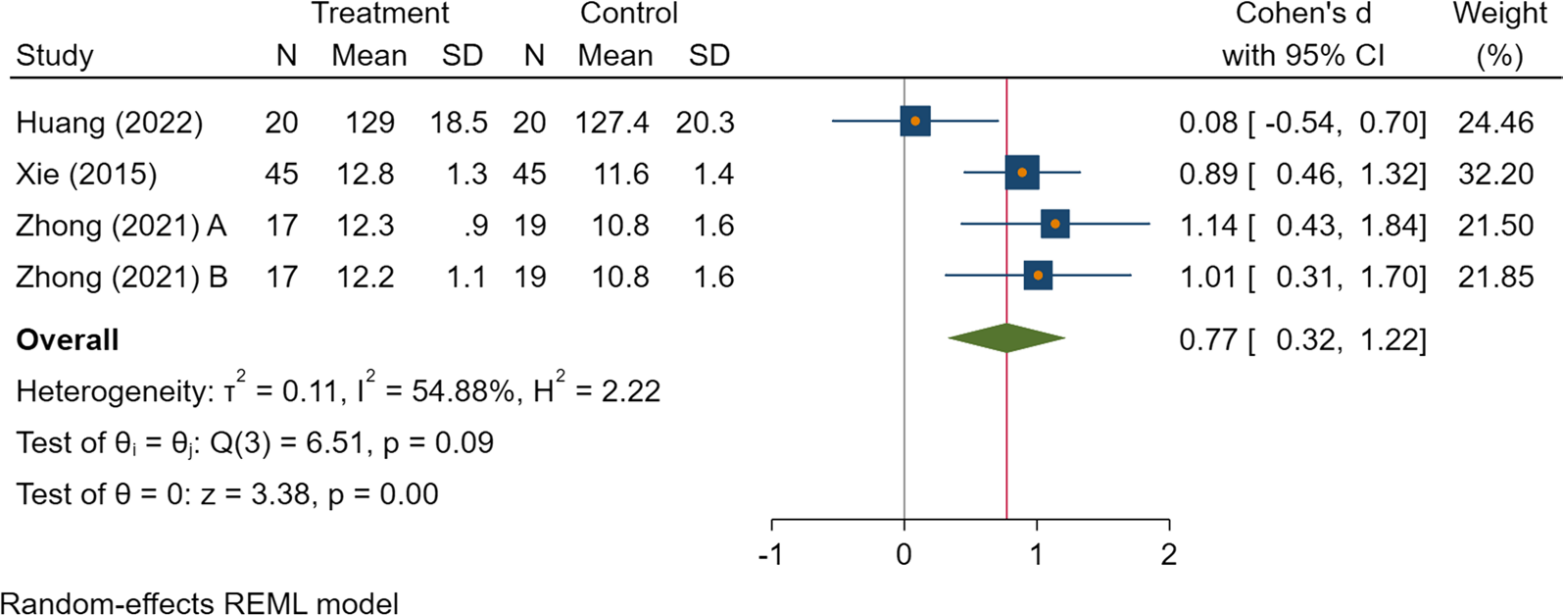
The forest plot on the level of postoperative hemoglobin

The rate of wound complications was lower in the TXA group than in the control group (Log OR = − 1.10 [95% CI − 2.17, − 0.02], *I*^2^ = 0%, moderate certainty of evidence) (Fig. 3).

### Other outcomes

**Fig. 4:**
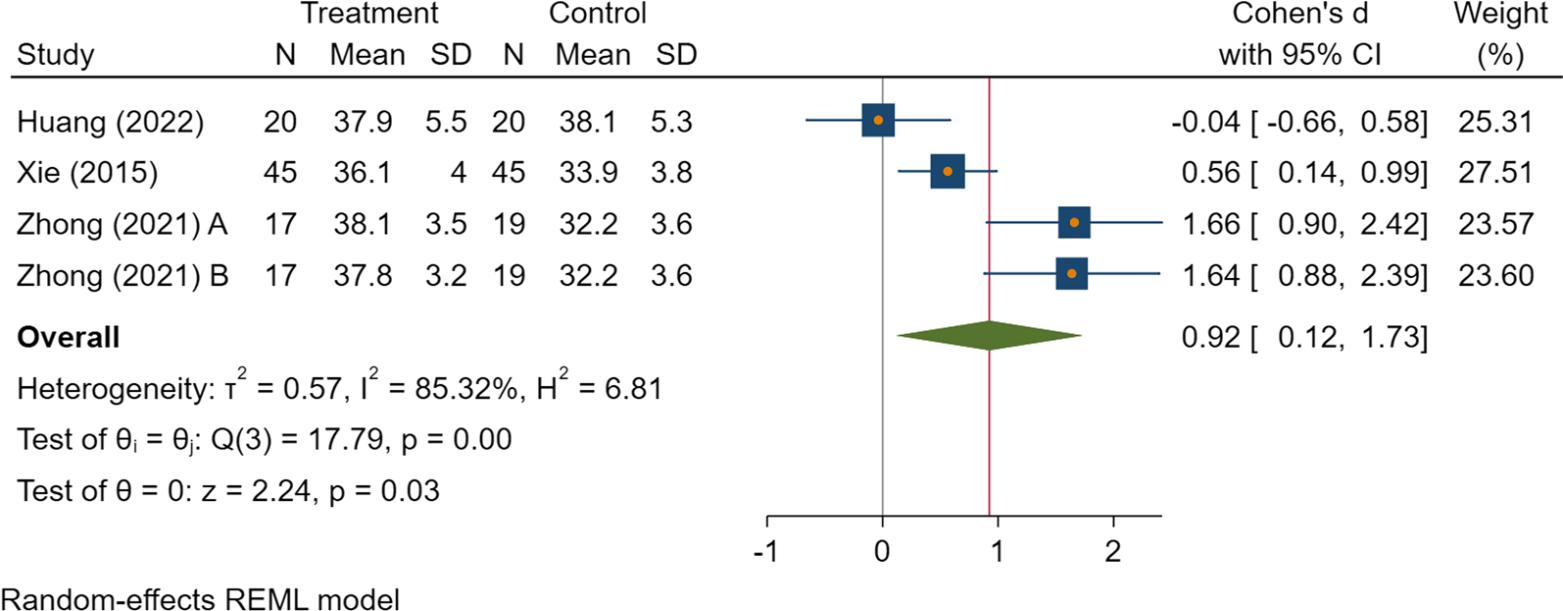
The forest plot on the level of postoperative hematocrit

**Fig. 5:**
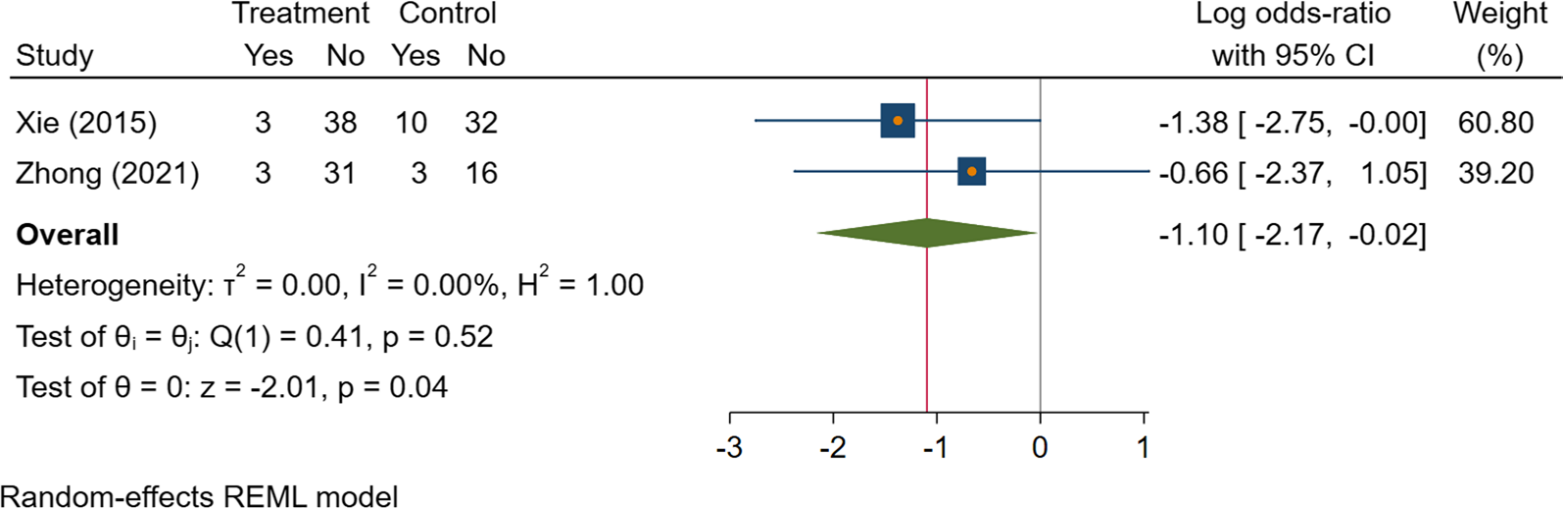
The forest plot on the rate of wound complications

As for other clinical outcomes, there was no difference in the hospital stay, as well as the duration of surgery. Outcomes of the blood test showed that TXA was associated with higher levels of hemoglobin (SMD = 0.77 [95% CI 0.32, 1.22], *I*^2^ = 54.88%, low certainty of the evidence) and hematocrit (SMD = 0.92 [95% CI 0.12, 1.73], *I*^2^ = 85.32%, low certainty of the evidence) (Figs. 4, 5). And there was no difference in the results of platelet count, activated partial thromboplastin time, and prothrombin time.

### Sensitivity

The “one removed” meta-analysis was performed by removing each individual study from the model, and there was no evidence that the removal of any single study resulted in a change in the conclusion that TXA does not reduce the postoperative drainage volume, the volume of intraoperative blood loss, the length of hospital stay, or the level of hematocrit.

### Publication bias

These results were consistent for both random effects and fixed effects statistical models, and we observed no evidence of publication bias, either when evaluating the funnel plot or statistically.

## Discussion

### Summary of main results

In this meta-analysis and systematic review, TXA administration did not reduce the volume of intraoperative blood loss but was associated with reduced blood loss during the first 24 h after surgery. And TXA improved the levels of hemoglobin, and hematocrit, and reduced the risk of wound complications. In view of the evidence, tranexamic can be safely used in calcaneal fractures patients and has the potential benefit of blood reduction. These findings are consistent with the growing body of evidence that demonstrates the safety and efficacy of TXA in surgeries.

### Blood loss

The surgical anatomy of the calcaneus is complex, with spongy cancellous bone and a rich blood supply. Calcaneus usually forms a cavity after fracture surgeries, resulting in significant blood loss despite open reduction and internal fixation, which may aggravate patients’ general conditions and increase the possibility of blood transfusion and infection [17, 18]. The reported postoperative blood loss of patients who had calcaneal fractures was around 300 ml [3, 4]. TXA is capable of binding to plasminogen and preventing tissue-type plasminogen activator (t-PA)-mediated release of active plasmin via the prevention of plasminogen binding to fibrin via lysine-binding sites [19, 20]. In our study, we found that the administration of TXA significantly reduced the postoperative blood loss (as we consider postoperative drainage can be regarded as invisible postoperative bleeding and confirmed the previous studies) in the first 24 h, whereas there was no difference in the total volume or those after 24 h. Our results are like previous studies [20–23], and relevant reasons are that TXA has a half-life of 2 h, and the effects are strongest in the first 24 h and begin to weaken over the subsequent time.

### Blood tests

Our study confirmed the hemostatic effect of TXA in calcaneal fracture surgeries and found a higher level of hemoglobin as well as hematocrit. These results also enhance the previous findings that the TXA could reduce blood loss, and our results agree with previous studies in orthopedic surgeries [1, 24].

Moreover, our results found similar levels of platelet count, prothrombin time (P.T.), and activated partial thromboplastin time (APTT) between the two groups, suggesting that the systematic coagulation was not affected by either local or intravenous administration of TXA. Our results were like previous studies. Possible reasons are the dosage used in our included studies was relatively small.

### Complications

Our study found that patients in the TXA group had statistically lower rates of wound complications, which may be associated with the reduced blood supply of the soft tissues after the use of TXA [1]. Another reason is the antifibrinolytic function of TXA (TXA could induce proinflammatory effects by activation of monocytes, neutrophils, platelets, endothelial cells, complement-releasing lipid mediators and cytokines, and induction of proinflammatory genes or proteins [25]). Of note, when TXA was given via topical method [13], wound complication was not reported, while in an intravenous way [14], the rate of postoperative wound complications was lower in the TXA group by 16.5%. Possible reason is that topical application of low-dose TXA (0.05 g) could decrease circumstance systemic absorption. Due to limited studies, meta-regression cannot be presented, but the way how TXA was given may exert an effect on the occurrence of wound complications [26, 27].

Theoretically, the use of TXA could potentially increase the incidence of thromboembolic events. Some investigators suggested that TXA activates fibrinolysis but does not affect coagulation. However, due to limited data, these outcomes could not be quantitatively synthesized. In the study of Xie et al. [4], there was no significant statistical difference between the TXA group and the non-TXA group. And in the study of Huang et al. [3], the systemic coagulation function of patients was not affected by TXA, and no thrombotic or cardiovascular event was found.

Despite the advances in non-operative and operative management, fractures of the calcaneus remain serious injuries that commonly affect young and active individuals and are often associated with long-term sequelae, permanent disability, a considerable reduction in quality of life and a high socio-economic cost [28]. Though our study found the benefit of TXA in the management of calcaneal fractures via surgeries, there is still a need for a carefully designed large-scale trial comparing surgery and non-operative management [29].

### The strengths and limitations of the review

The strengths of this meta-analysis include (i) the clear definition of the research question used in this study to reduce bias in the selection of studies, (ii) adherence to an explicit research protocol that was developed prior to the analysis, (iii) comprehensive literature search and the use of consensus reached by two reviewers in the selection of articles, and (iv) a quality control review of all the results. The included studies in this meta-analysis were RCTs.

However, this meta-analysis also has some limitations. First, though the use of TXA was proven to be safe and effective in our study, the most appropriate dose was not investigated. Moreover, how the dosage, duration, number of dosages, and time of administration could influence the results have not been investigated due to limited studies. Second, the sample size was not large. Subgroup analysis and meta-regressions are needed to find more information. The body of evidence for TXA use in calcaneal fracture surgeries has not grown as rapidly as in arthroplasty surgery. In light of the preliminary favorable results of our study, we call for a larger sample size and a multicenter study to investigate the effect and safety of TXA in calcaneal fracture surgeries.

## Conclusions

In conclusion, TXA significantly reduced blood loss during the first 24 h postoperatively, improved the levels of hemoglobin and hematocrit, and reduced the risk of wound complications. In view of the evidence, TXA can be safely used in patients with calcaneal fractures and has the potential benefit of blood reduction.

## Data Availability

The datasets generated and/or analyzed during the current study are not publicly available due to techniques but are available from the corresponding author on reasonable request.

## Abbreviations

TXA: Perioperative tranexamic acid
RCTs: Randomized controlled trials
PRISMA: Preferred Reporting Items for Systematic Reviews and Meta-analyses
GRADE: Grading Recommendations Assessment, Development, and Evaluation
SMD: Standard mean difference
RRs: Relative risks
95% CI: 95% Confidence intervals
